# Research on Medical Image Super-Resolution Reconstruction Algorithm Based on Dilated Convolution and Multi-Module Fusion

**DOI:** 10.3390/jimaging12080391

**Published:** 2026-08-19

**Authors:** Zhuye Xu, Yucong Guo

**Affiliations:** School of New Energy and Power Engineering, Lanzhou Jiaotong University, Lanzhou 730070, China; xuzhuye@mail.lzjtu.cn

**Keywords:** super-resolution reconstruction, dilated convolution, attention mechanism, deep learning

## Abstract

Medical image resolution plays a crucial role in early disease detection and fine-structure observation. Super-resolution reconstruction technology can restore low-resolution images to high-resolution versions, thereby assisting physicians in making accurate diagnoses. To address challenges in medical image super-resolution reconstruction, including insufficient global information acquisition, excessive network complexity, and suboptimal loss function adaptation for medical imaging data, this paper proposes an image super-resolution reconstruction algorithm named IDCASR-MMF based on improved dilated convolution and multi-module fusion. First, multi-dilation-rate dilated convolution is introduced to expand the receptive field and integrated with a spatial attention mechanism to dynamically calibrate high-frequency features after feature extraction. Subsequently, the Squeeze-and-Excitation module is fused with dilated convolution as a channel attention mechanism to streamline the network architecture. Finally, a weighted fusion strategy combining adversarial loss and MSE loss is adopted, where the dynamic adjustment of weighting coefficients balances pixel-level structural accuracy and high-frequency detail authenticity, achieving synergistic optimization of objective precision and subjective quality for medical images. To validate the effectiveness of the proposed algorithm, IDCASR-MMF is compared with 11 state-of-the-art methods across five datasets (Set5, Set14, BSD100, Urban100, and Bone FD). Experimental results demonstrate that the proposed algorithm achieves superior PSNR and SSIM values on multiple datasets, confirming that IDCASR-MMF can effectively reconstruct high-resolution medical images from low-resolution inputs.

## 1. Introduction

Medical imaging serves as an indispensable visualization tool in clinical diagnosis and treatment, enabling clear visualization of internal physiological structures and pathological features within the human body. It holds critical value for early lesion screening, surgical planning, and therapeutic efficacy assessment [1,2,3]. However, limitations in physical imaging device resolution [4], patient motion artifacts, and clinical demands for rapid imaging protocols [5] often result in insufficient spatial resolution of acquired images. This deficiency leads to blurred key details such as microcalcifications and microvascular branches [6], directly compromising diagnostic accuracy in tumor margin delineation and microstructural anomaly identification [7]. To address these challenges, medical image super-resolution reconstruction (MISR) technology has emerged. By restoring low-resolution (LR) images to high-resolution (HR) spatial domains, MISR significantly enhances image detail characterization capabilities, providing reliable support for precision diagnostics and treatment planning [8,9].

The theoretical foundation of super-resolution technology can be traced back to the alternating projection algorithm proposed by Gerchberg and Saxton in 1972 [10]. By iteratively optimizing spatial and frequency domain constraints, this algorithm mathematically validated the feasibility of reconstructing high-resolution information from low-resolution observational data for the first time, providing theoretical support for overcoming the optical diffraction limit. Early traditional methods primarily relied on multi-frame image registration and manual verification but faced significant limitations in practical applications [11]: their generalization performance drastically deteriorates when handling complex noise interference and motion blur scenarios. Particularly in medical imaging, the reconstruction error rate for micron-level pathological features can reach 37%, making these methods inadequate for clinical precision diagnostics.

In medical imaging applications, the rapid advancement of deep learning technologies has demonstrated dual technical advantages in super-resolution reconstruction [12]. Firstly, hierarchical nonlinear transformation networks enable simultaneous modeling of low-order statistical features and high-order semantic correlations in images. Secondly, these systems exhibit adaptive capabilities across multi-device and degradation scenarios, autonomously learning from millions of medical imaging datasets without requiring predefined models. These technological breakthroughs are driving the transition of medical image analysis from traditional isolated processing to intelligent full-process workflows, establishing super-resolution as a cornerstone technology in precision medicine ecosystems. In 2014, Dong et al. proposed SRCNN [13], the first end-to-end low-resolution-to-high-resolution nonlinear mapping model using a three-layer convolutional neural network, achieving a peak signal-to-noise ratio improvement and 12× acceleration in natural image super-resolution tasks. Building upon this, Kim’s team developed two influential deep learning architectures: First, VDSR [14] innovatively introduced residual learning mechanisms by establishing residual relationships between low-resolution inputs and target images. This approach not only surpassed SRCNN’s depth limitations but also effectively mitigated gradient instability in deep network training. Their subsequent work, DRCN [15], implemented parameter sharing through recursive structures, constructing deep networks via multi-level recursive operations within single convolutional layers, while expanding the receptive field through residual learning strategies. Together, these methods advanced deep learning applications in super-resolution through dual pathways: network depth expansion and parameter optimization.

In 3D medical imaging, Pham’s team [16] drew inspiration from SRCNN to pioneer a 3D convolutional neural network architecture. By leveraging multi-layer feature extraction through 3D convolution kernels, their model achieved voxel-wise resolution enhancement in brain tissue MRI scans. Addressing gradient decay during network training, Shi’s research group [17] devised a multi-level residual optimization framework, innovatively combining joint training of local detail refinement and global structural correction. This dual-path residual mechanism demonstrated exceptional edge preservation capabilities in MRI reconstruction. Notably, Chen et al. [18] proposed DCSR, a 3D densely connected network employing high-density cross-layer connections. By creating inter-layer feature reuse pathways, DCSR significantly improved texture feature transmission efficiency in brain MRI, achieving groundbreaking performance in reconstructing fine structures like gray-white matter boundaries.

Despite the remarkable advantages of deep learning in medical image super-resolution tasks, three critical limitations persist: insufficient global context modeling, the trade-off between computational efficiency and model performance, and loss function designs inadequately adapted to medical imaging characteristics. To address the deficiency in global context modeling, Li et al. [19] proposed a multi-scale residual network for image super-resolution reconstruction, but its convolutional kernels fail to effectively model cross-region dependencies. The DRCN model employs a deeply recursive convolutional architecture with up to 16 recursive layers, enhancing large-range context utilization through recursive supervision and skip connections, yet it overlooks high-frequency components (e.g., edges and textures) in low-resolution images’ multi-scale features. Regarding the efficiency-performance balance, mainstream models like EDSR [20] and RCAN [21] rely on deep stacking to boost performance, resulting in excessive parameter counts (43 M and 16 M, respectively), which hinder real-time intraoperative applications. Although lightweight designs such as Lu et al.’s SG-SRNet [22] reduce parameters to 4.3 M via channel pruning, over-compression risks losing subtle lesion features critical for diagnosis. For loss function adaptation, Wang et al.’s MSANet [23] incorporates pyramid pooling to aggregate multi-level features but suffers from limited small-lesion detection (78.5% accuracy) due to fixed-dilation-rate convolutions. These limitations collectively impede clinical adoption. Additionally, Zhao et al. [24] demonstrated that while the MSE-trained ProSR model achieves a 1.2 dB PSNR improvement in lung nodule CT reconstruction, edge sharpness metrics degrade by 19%, and the multi-scale properties of medical images remain insufficiently captured [25]. To overcome these limitations, generative adversarial networks have been widely introduced to enhance high-frequency textures and edge sharpness [26].

To address the aforementioned challenges, this paper proposes IDCASR-MMF (Improved Dilated Convolution Attention–Multiple Module Fusion), a medical image super-resolution reconstruction network integrating multi-scale dilated convolution and hybrid loss functions. The method employs multi-scale dilated convolutions to expand the receptive field, enhancing global and local feature modeling capabilities, while incorporating spatial and channel attention mechanisms to better capture subtle lesions and high-frequency details. Simultaneously, a hybrid loss function balances pixel-level accuracy and high-frequency detail recovery, mitigating over-smoothing caused by traditional MSE loss. With parameter count reduced to 8.7 M, our approach achieves real-time super-resolution reconstruction of medical imaging data, providing high-precision visual support for medical navigation and AI-assisted diagnosis. Key innovations include the following:

(1) Multi-scale dilation attention module: Design parallel dilation volume integration branch with differential expansion rate and capture local details and global anatomical correlation through the hollow space pyramid; a dynamic channel calibration mechanism was introduced to enhance the ability to focus on pathological features such as microcalcifications according to the significance of the lesion area.
(2) Design a novel composite loss optimization strategy: To address the training instability and mode collapse inherent in standard GANs, we propose a dynamic scheduling mechanism based on multi-stage warm-up and linear scaling, tightly coupled with a cosine annealing learning rate strategy. This mechanism adaptively schedules the weights of the MSE and adversarial losses across different training stages, synergistically optimizing high-frequency detail recovery while strictly preserving the underlying anatomical structural fidelity.

## 2. Materials and Methods

### 2.1. IDCASR-MMF Monolithic Framework

**Figure 1:**
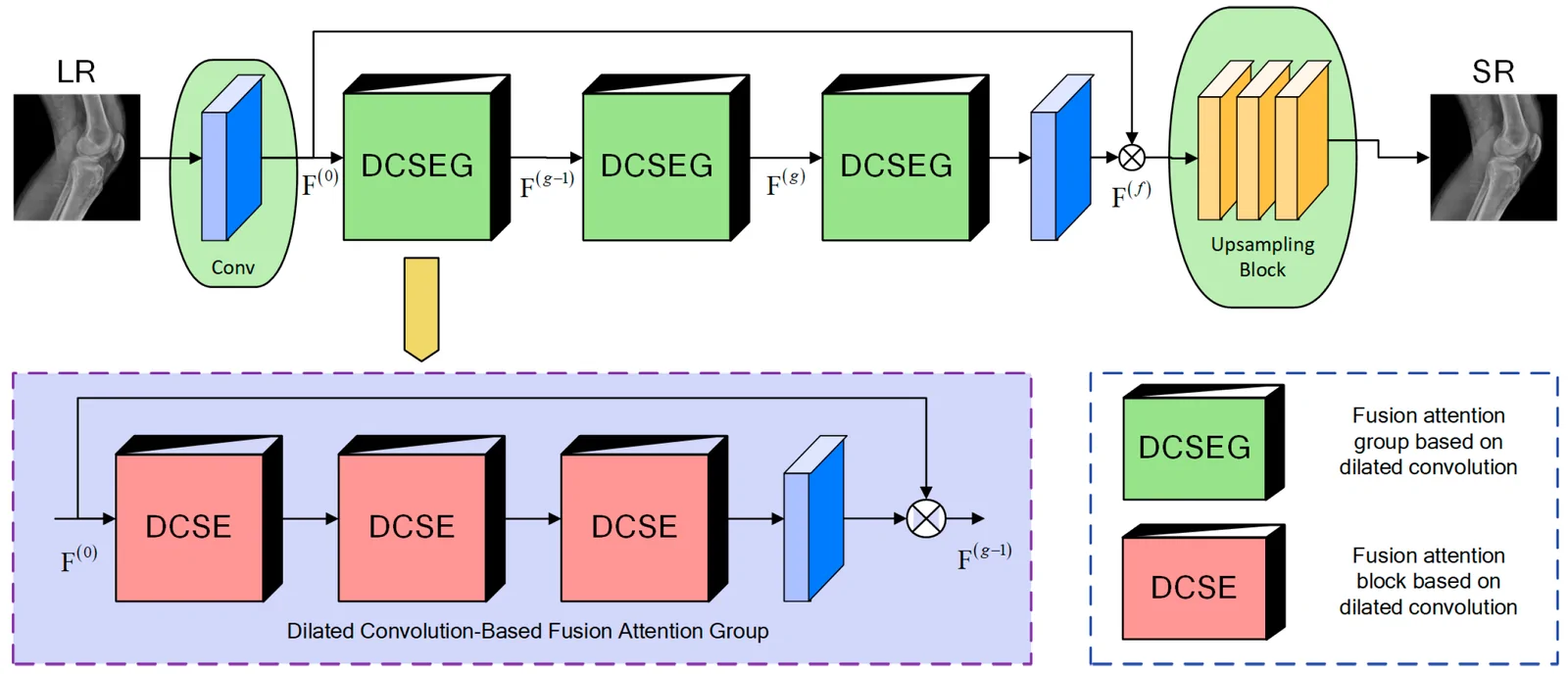
Fusion Attention Network IDCASR-MMF Model Framework Based on Hole Convolution.

In order to obtain more high-frequency components of the multi-scale context information of low-resolution images in the process of super-resolution reconstruction of medical images, and then improve the performance of image super-resolution reconstruction, this paper proposes an image super-resolution reconstruction model that integrates the Squeeze-and-Excitation (SE) channel attention mechanism with dilated convolutions. The overall framework of IDCASR-MMF is illustrated in Figure 1.

Specifically, the multi-scale dilated convolutions are utilized to capture the long-range contextual dependencies within the image. Subsequently, a dual attention mechanism, which integrates both spatial and channel (SE) attention, adaptively recalibrates the extracted multi-level feature representations across spatial regions and feature channels. In addition, this study also improves the traditional MSE loss function and proposes an edge retention weight adjustment strategy based on the mixed loss of Adversarial Loss and Mean Square Error Loss (MSE Loss). This method can not only effectively balance the detail recovery and the overall quality of the image structure but also better focus on the region of interest and improve the recovery effect of important features in low-resolution images through the introduction of attention mechanism.

As can be seen in Figure 1, IDCASR-MMF first converts the low-resolution image into a primary feature representation through the convolution operation of the input layer, which is the basic feature extraction stage of the entire super-resolution network. In this process, the spatial perception ability of the convolutional kernel is used to capture the local texture pattern of the image, and the original pixel space is mapped to the high-dimensional feature space. The initial feature map $F^{\left(0\right)}$ not only retains the low-frequency structure information of the input image but also provides an extensible feature base for the subsequent depth feature transformation. The specific performance formula is as follows:

(1) $$F^{\left(0\right)}=H_{conv}\left(LR\right)$$

where $H_{conv}$ stands for convolution operation. After the initial feature extraction, the information will be transmitted to the Fusion Attention Group (DCSEG) based on dilated convolution, and each DCSEG module realizes iterative optimization of features through the residual structure, and the nested multi-branch dilated convolutional network inside it constructs a multi-scale feature perception system. The dilated convolutional layer expands the receptive field while maintaining the efficiency of the parameters through different expansion rates, effectively capturing the contextual associations across different spatial distances in the image.

The attention mechanism introduced in the feature fusion stage dynamically calibrates the feature response of each channel, and strengthens the expression ability of important feature channels through global average pooling and weight vectors generated by the fully connected layer. The combination of this residual connection and attention mechanism enables the network to avoid gradient attenuation and adaptively focus on the recovery of high-frequency details in the deep training process, forming a progressive optimization path in the feature space. The specific performance formula is as follows:

(2) $$F^{\left(g\right)}=F^{\left(g-1\right)}+H_{DCSEG}^{\left(g\right)}\left(F^{\left(g-1\right)}\right),g=1,2,\ldots ,G$$

$H_{DCSEG}$ means that the convolution operation is carried out inside the DCSEG module, and after the processing of all DCSEG modules is completed, the network aggregates the initial features and the intermediate features of each stage through hopping connections. This cross-layer feature fusion mechanism realizes the complementarity of features at different levels of abstraction, which not only retains the underlying texture features captured by the shallow network but also integrates the complex semantic information extracted by the deep network. The final feature map is constructed by linear overlay $F^{\left(f\right)}$. In essence, a composite expression space containing multi-scale and multi-receptive field features is constructed, which effectively overcomes the problem of layer-by-layer attenuation of feature information in the traditional cascade structure, and provides an information-complete feature substrate for subsequent upsampling and reconstruction. The specific performance formula is as follows:

(3) $$F^{\left(f\right)}=F^{\left(0\right)}+\sum_{g=1}^{G}F^{\left(g\right)}$$

Finally, the global fusion features are reconstructed from high-dimensional feature space to high-resolution pixel space through the upsampling module composed of sub-pixel convolution or transpose convolution. While expanding the spatial dimension of the feature map, the upsampling layer maintains the continuity of high-frequency details through the recombination calculation of multi-channel features, and finally outputs a super-resolution image with significantly improved texture clarity and edge sharpness. This end-to-end reconstruction mechanism unifies feature optimization and spatial transformation under the deep learning framework to achieve pixel-level precision image restoration.

### 2.2. Dilated Convolutional Attention Module

The Dilated Convolution Attention Module is an innovative module that combines convolutional neural networks (CNNs) and attention mechanisms, which is mainly used to improve the performance of neural networks when dealing with tasks with long-range dependence [25]. This convolution module is a special convolution operation that expands the receptive field by inserting holes (i.e., spacing) between elements in the convolutional kernel, making it possible to capture a larger range of contextual information while keeping the amount of computation and parameters low. Compared with traditional convolution operations, dilated convolution is able to capture more region information with a smaller convolution kernel. For example, a traditional convolution operation would be

(4) $${\left(I^{*}\varpi \right)}_{\left(t\right)}=\sum_{p+q=t}I_{\left(p\right)}\varpi_{\left(q\right)}$$

where I represents the input of the convolutional layer and ϖ represents the convolutional kernel. The operation of dilated convolution is

(5) $${\left({I^{*}}_{l}\varpi \right)}_{\left(t\right)}=\sum_{p+lq=t}I_{\left(p\right)}\varpi_{\left(q\right)}$$

where l is the dilation rate. Compared with the traditional convolution operation, the void convolution operation can be carried out in different ranges through different dilation rates, so as to expand the receptive field without adding additional computational complexity.

However, there are limitations to this method of expanding the size of the receptive field by using a variety of convolutional kernels of different sizes and multi-branched structures: although the large convolutional kernel can increase the receptive field, the surge of parameters will lead to low computational efficiency and increased training complexity, and the multi-branched structure will also increase the risk of model overfitting. However, although the small convolutional kernel is conducive to lightweight, it is limited by the range of local feature perception, which makes it difficult to effectively model multi-scale context information, and ultimately restricts the performance of the model in complex scenes.

**Figure 2:**
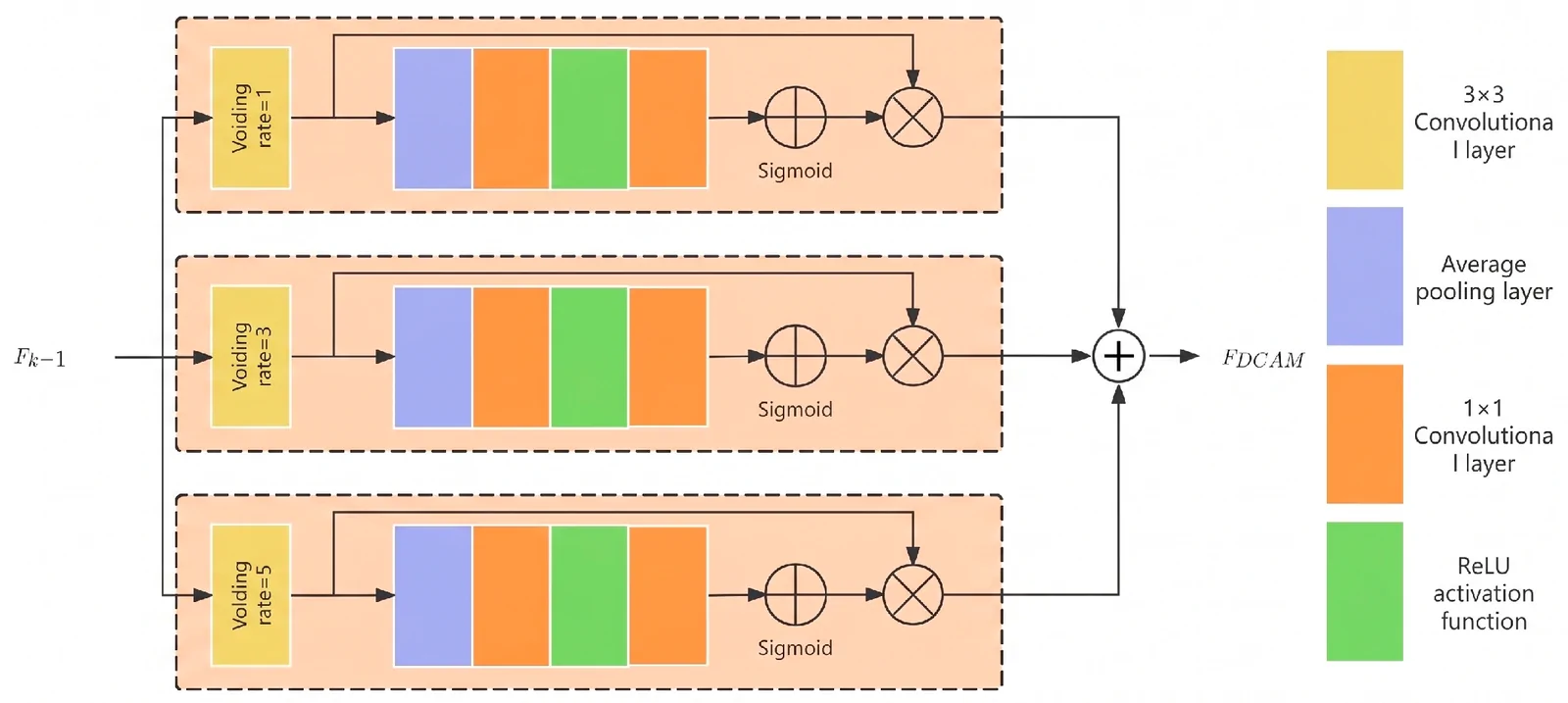
Diagram of the structure of Dilated Convolutional attention.

Therefore, this paper comprehensively considers the memory requirements of the model and the information requirements of capturing features when designing the dilated convolutional attention module, and the specific structure of the final design is shown in Figure 2.

As shown in the figure, the dilated convolutional attention module in this paper has three different attention blocks, and the dilated convolutional attention module captures multi-scale context information in multiple different regions through three empty attention blocks with different size receptive fields. Each hole attention block consists of a hole convolutional layer and a channel attention. In the actual operation process, the dilated attention block first extracts the features of the fixed receptive field area in the low-resolution image with the help of a dilated convolutional layer with a set void rate. The features extracted by this process cover both low-frequency and high-frequency features. Secondly, the average pooling layer is used to process the features just extracted, and the corresponding statistics are obtained from them. Contextual information is captured through a structure composed of convolutional layers, ReLU activation functions, and convolutional layers. Finally, the Sigmoid activation function was used to recalibrate the previously extracted features in combination with the channel multiplication operation. The main purpose of this step is to allow the model to focus on high-frequency features to optimize the processing and analysis of image information. The specific calculation process is as follows:

(6) $$\hat{X}=\sigma \left(f_{c_{2}}\left(\delta \left(f_{c_{1}}\left(pool_{avg}\left(X\right)\right)\right)\right)\right)⊗X^{\prime }$$

where $X^{\prime }$ represents the input feature of the empty attention block; $\hat{X}$ represents the output feature of the empty attention, the characteristic of recalibration; σ stands for Sigmoid activation function operation; δ represents the ReLU activation function operation; $pool_{avg}\left(X\right)$ represents the average pooling layer operation; and $f_{c_{1}}f_{c_{2}}$ represents an operation with 2 convolutional layers.

In the void convolutional attention module, the void rates of three different void attention blocks are set to 1, 3, and 5, respectively, and the corresponding receptive field sizes are 3, 11, and 19, respectively. When the three void attention fields have different receptive fields, they can obtain more low-resolution image features and detail information in multiple different regions, so that the dilated convolutional attention module can capture multi-scale context information in multiple different regions of the low-resolution image.

### 2.3. Dual Spatial and Channel Attention Mechanism

To dynamically model the dependencies across channel dimensions, this paper incorporates the Squeeze-and-Excitation module [27] to calibrate the feature maps. This module first extracts global spatial information per channel via global average pooling, then generates adaptive channel weights using a two-layer fully connected network. Finally, these weights are applied to recalibrate each channel, effectively amplifying salient features while suppressing irrelevant noise. This mechanism can significantly improve the feature expression ability of the network, and at the same time, the computational overhead is low, which is suitable for the task of super-resolution reconstruction of lightweight images.

**Figure 3:**
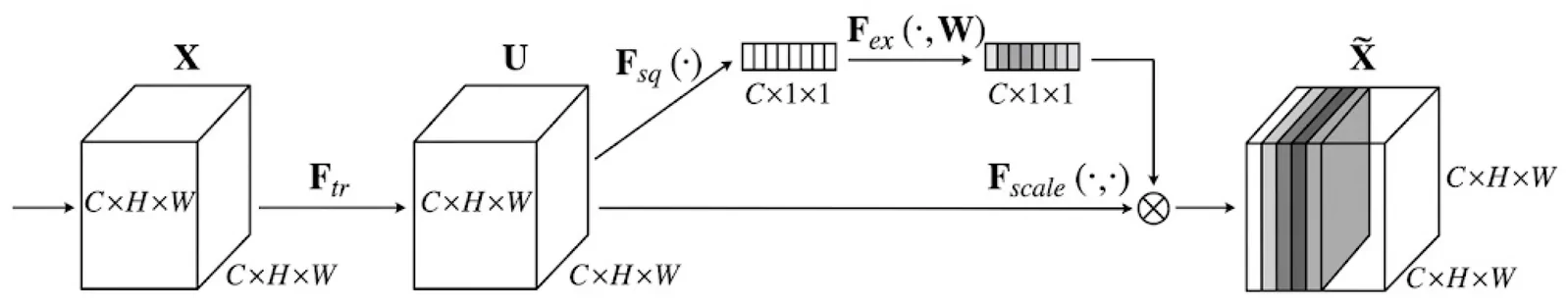
SE Attention Module Structure Diagram.

The SE module consists of three main steps: Squeeze, Excitation, and Reweight. The specific structure of the design is shown in Figure 3.

The first step is Squeeze, which calculates the global feature statistics of each channel by pooling the average of the channel, compresses the input feature map into a global description, and then extracts the global information from the spatial dimension, as shown in Equation (7):

(7) $$z_{c}=\frac{1}{H\times W}\sum_{i=1}^{H}\sum_{j=1}^{W}F\left(i,j,c\right)$$

The second step is Excitation, which learns the dependencies between channels through a fully connected network and generates the weights for each channel, mathematically, as follows:

(8) $$s=\sigma \left(W_{2}\delta \left(W_{1}z\right)\right)$$

The third step is Reweight, which readjusts the weight of each channel obtained after feature excitation to the importance of each channel of the input feature map, and multiplies the weights with the input features element by channel.

In summary, we can get the core formula of the SE module as follows:

(9) $$F^{\prime }=F\cdot \sigma \left(W_{2}\delta \left(W_{1}GAP\left(F\right)\right)\right)$$

where GAP(F) is the global average pooling of input feature graph F for obtaining the channel description, $W_{1}$ and $W_{2}$ are the weight matrices of a two-layer fully connected network, δ is the ReLU activation function, and σ is the Sigmoid activation function.

The advantages of the SE module are its lightweight design and versatility, which significantly improves the feature expression ability of the network through global average pooling and a two-layer fully connected network, while introducing a small number of additional calculations and parameters. And it can be seamlessly embedded in various convolutional neural networks, and has excellent performance in tasks such as image classification, object detection, and super-resolution reconstruction, which is an efficient and widely applicable attention mechanism.

To complement the channel-wise recalibration of the SE module, a spatial attention branch is computed in parallel. It utilizes 1×1 convolutions to aggregate cross-channel information, followed by a Sigmoid activation to generate a spatial attention map. This allows the network to explicitly focus on informative high-frequency regions, such as micro-lesions.

### 2.4. DCSE Module

**Figure 4:**
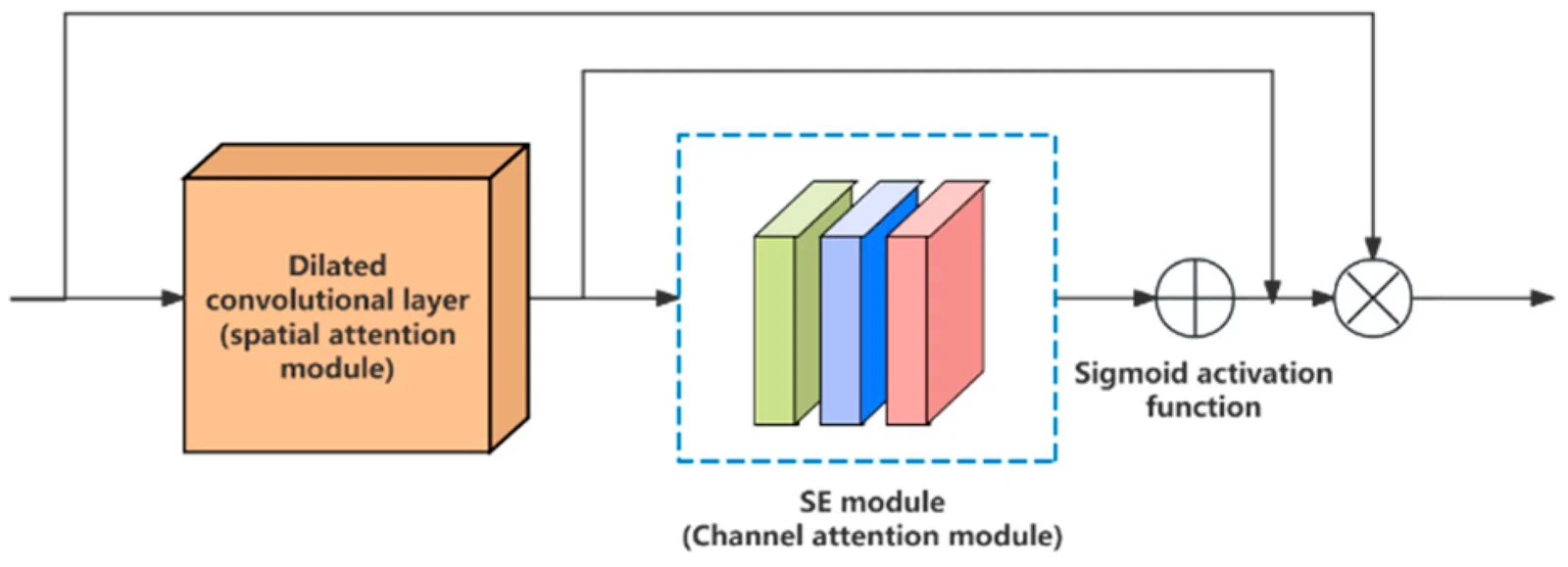
Fusion Attention Block (DCSE) structure diagram based on dilated convolution.

In this paper, we design an attention mechanism called DCSE (Dilated Convolution with Squeeze-and-Excitation), which is composed of three parts, namely a branched dilated convolutional layer, channel attention gating unit, and residual connection, and the deep integration of multi-scale dilated convolution and channel attention mechanism realizes the adaptive feature reinforcement of image local details and global semantic information. DCSE can not only use the dilated convolution to expand the receptive field to capture the dependence of long-distance but also adaptively adjust the channel importance of the feature map through the SE module, so as to further enhance the representation ability of the model. The DCSE structure is shown in Figure 4.

In terms of macro architecture, the DCSE module constructs a differentiated receptive field through parallel dilated convolution branches and uses convolutional kernels with different expansion rates to synchronously extract multi-scale features of images, and its specific dilation rate is described in 2.2 of this paper. Among the different attention blocks designed in this paper, the low-dilatation branch focuses on pixel-level high-frequency details, while the high-dilatation branch captures a larger range of contextual associations. This multi-scale design breaks through the limitations of the receptive field of traditional single-branch convolution and avoids the loss of spatial information caused by continuous downward sampling. At the same time, the module decouples spatial feature extraction from channel dimension mapping, which significantly reduces the computational cost and achieves a balance between lightweight and high efficiency.

After the features are extracted by the void convolutional layer, the SE module introduces the channel attention mechanism to dynamically calibrate the multi-branch output. The channel-level statistical features are obtained by compressing the spatial dimension of global average pooling, and then the channel weight vector is generated by the fully connected layer and nonlinear activation. The mechanism can autonomously identify the feature channels that are sensitive to the current task and suppress the channel responses of redundancy or noise interference, so as to complete the adaptive enhancement and suppression before feature fusion, and improve the semantic directivity of feature expression.

As outlined in the algorithm pseudocode, the DCSE module applies the spatial and channel attention through a sequential composition. Given the concatenated multi-scale feature map X, the channel attention vector $Y\in R^{B\times C\times 1\times 1}$ and the spatial attention map $W_{s}\in R^{B\times 1\times H\times W}$ are computed, respectively. The composite feature recalibration is defined as

(10) $$X_{c}=X⊗Y$$

(11) $$X_{out}=X_{c}⊙W_{s}$$

where ⊗ denotes channel-wise broadcasting multiplication, and ⊙ represents spatial element-wise multiplication. By composing them, the module simultaneously filters redundant channels and highlights critical spatial structures.

Eventually, the calibrated multiscale features are fused by additive element by element and residuals connected to the original input passed by the jump link. This dual-path design not only retains the low-level visual cues of the shallow network but also strengthens the high-order semantic information of the features through deep nonlinear mapping. The residual structure not only alleviates the gradient dispersion problem but also gradually increases the energy proportion of high-frequency details through layer-by-layer iterative optimization, so that the key structures of the reconstruction target can be accurately represented in the feature space. On the whole, the DCSE module achieves a breakthrough in computational efficiency and feature expression ability through the synergy of multi-scale, dynamic calibration and residual learning, so that the memory requirement of the overall model is only 8.7 M, which lays a key technical foundation for low-computational, high-precision image reconstruction tasks. 

The overall structure of the above model can be summarized by pseudo-codes, as shown in Algorithm 1.
**Algorithm 1.** Pseudo-Code Display of DCASR Attention Mechanism, Algorithm DCASR Attention Mechanism Pseudocode1. Input: Feature map X, Compression ratio r, Dilation rate set D = {d_1_,d_2_,…,d_k_} Output: Enhanced feature map X_out_ ∈ R^{B×C×H×W}^2. Spatial attention computing: For d ∈ D:  F_d ← DepthwiseConv3 × 3(X_c; padding = d, dilation = d) ∈ R^{B×C×H×W}^ F ← Concat(F_d_1_,…,F_d_k_) ∈ R^{B×(k·C)×H×W}^ F ← Conv1 × 1(F) → BatchNorm → ReLU W_s ← Conv1 × 1(F) → Sigmoid3. Channel attention calculation: Y ← GlobalAvgPool(X) ∈ R^{B×C}^ Y ← FC(C→C/r)(Y) → ReLU → FC(C/r→C)(Y) → Sigmoid ∈ R^{B×C}^ X_c ← X ⊗ Y.view(B,C,1,1)4. Fusion and output X_out_ ← X_c ⊙ W_s ∈ R^{B×C×H×W}^5. Remark Conv1 × 1: 1 × 1 convolution changes the number of channels DepthwiseConv3 × 3: Depth-separable convolution

### 2.5. Mixed Loss Function

Adversarial Loss and Mean Square Error Loss (MSE Loss) are two widely used loss functions in deep learning image generation and super-resolution tasks, which constrain the generative network at different levels to improve image quality. However, there are significant drawbacks to using either loss function alone. Therefore, this paper proposes a new composite loss function improvement strategy that combines the advantages of the two, aiming to balance pixel-level accuracy and perceptual quality.

Adversarial Loss is the core loss function of the Generative Adversarial Network (GAN), which is directly derived from the training target of the GAN. In GAN, the game between the generator and the discriminator is played through adversarial training, which improves the quality of the image generated by the generator. The goal of the discriminator is to distinguish between the real image and the generated image, so the discriminator is calculated from the predicted value of the real image and the generated image, as shown in the below formula:

(12) $$L_{D}=-E_{X_{HR}∼P_{HR}}\left[\log D\left(X_{HR}\right)\right]-E_{X_{LR}∼P_{LR}}\left[\log\left(1-D\left(G\left(X_{LR}\right)\right))\right)\right]$$

where $D\left(X_{HR}\right)$ represents the probability that the discriminator judges the ground-truth high-resolution image $X_{HR}$ as real, $G\left(X_{LR}\right)$ is the super-resolved image generated from the low-resolution input $X_{LR}$, and $P_{HR}$ and $P_{LR}$ denote the data distributions of the real high-resolution images and low-resolution inputs, respectively.

The goal of the generator is to reconstruct a realistic, high-resolution image from a low-resolution input. In super-resolution tasks, the generator is typically a deep convolutional neural network whose target is to produce results that are visually indistinguishable from real images. Therefore, the generator loss is defined as

(13) $$L_{G}=-E_{X_{LR}∼P_{LR}}\left[\log D\left(G\left(X_{LR}\right)\right)\right]$$

where $D\left(G\left(X_{LR}\right)\right)$ denotes the discriminator’s output, indicating the probability that the generated image is judged as real. The generator aims to maximize this probability to deceive the discriminator, effectively minimizing the corresponding negative log-likelihood.

To structurally realize the aforementioned adversarial feedback, the discriminator is engineered as a PatchGAN architecture. Rather than outputting a global scalar, it maps the input to an M×M matrix to evaluate local patch authenticity. Consequently, the theoretical adversarial objectives are executed as a spatial average loss across these local receptive fields:

(14) $$L_{D_{patch}}=-\frac{1}{M^{2}}\sum_{i=1}^{M}\sum_{j=1}^{M}\left[\log\left(D{\left(X_{HR}\right)}_{i,j})+\log\left(1-D{\left(G\left(X_{LR}\right)\right)}_{i,j})\right)\right)\right]$$

Topologically, this discriminator is constructed as a five-layer fully convolutional network. The first four layers employ 3×3 convolutional kernels with a stride of 2 for progressive spatial downsampling, utilizing LeakyReLU activations and Instance Normalization. The final layer directly outputs the unnormalized M×M matrix. During optimization, the discriminator operates with a fixed learning rate of $5\times 10^{-5}$ and maintains a strict 1:1 gradient update ratio with the generator.

While the adversarial penalty enhances high-frequency micro-textures, the Mean Squared Error (MSE) loss is simultaneously employed to guarantee macroscopic pixel-level fidelity. As defined in the super-resolution context, let $X_{LR}$ denote the low-resolution input and $X_{HR}$ the corresponding ground-truth high-resolution image. The MSE loss explicitly measures the pixel-wise discrepancy between the super-resolved output $G\left(X_{LR}\right)$ and the real $X_{HR}$, formulated as

(15) $$L_{MSE}=\frac{1}{N}\sum_{i=1}^{N}{\left(X_{HR}\left(x_{i}\right)-G\left(X_{LR}\right)\left(x_{i}\right)\right)}^{2}$$

Among them, $L_{MSE}$ is the mean square error loss value, which measures the pixel-level difference between the generated image and the real high-resolution image; N is the number of pixels involved in the calculation. The value of the $X_{HR}\left(x_{i}\right)$ represents the true high-resolution image at the i-th pixel; the $G\left(X_{LR}\right)\left(x_{i}\right)$ generator network G predicts the value of a super-resolution image generated from a low-resolution input image at the i-th pixel. Optimizing this loss function makes the pixel value of the generated image as close as possible to the real image. However, because MSE loss focuses on pixel-level precision, the resulting image may be very precise in detail but may lose texture and naturalness in perceived quality.

In the image super-resolution task, the generative adversarial network (GAN) optimizes the image perception quality through the adversarial game between the generator and the discriminator, and its unsupervised characteristics support the potential of cross-modal synthesis. However, GANs face core problems such as unstable training, limited evaluation metrics, and gradient vanishing due to the over-optimization of discriminators. Although the traditional MSE constrains pixel-level errors, it relies too much on low-order statistical features, resulting in the loss of high-frequency details and structural semantic distortion of the generated images. In particular, in the field of medical images, the above shortcomings are further highlighted: the micropathological structures are easy to blur under the constraints of MSE, and the high-frequency textures generated by GAN may deviate from the real anatomical features; in addition, the strict requirements of spatial resolution and structural fidelity of medical images contradict the lack of higher-order semantic constraints in existing models, making is difficult to meet the reliability standards of clinical diagnosis.

In order to overcome the above problems, this study proposes a complex optimization strategy that combines adversarial loss and MSE loss (Adversarial-MSE loss). On the one hand, the MSE loss constraint generates a pixel-level error between the image and the real image to ensure the accuracy of the basic structure and low-frequency details. On the other hand, the adversarial loss learns the real data distribution through the dynamic feedback-driven generator of the discriminator, which strengthens the high-frequency texture, edge sharpness and natural expression of the image. The synergy between the two allows the model to strike a balance between pixel-level fidelity and perceived quality—MSE loss inhibits the over-smoothing of the generated results, while adversarial loss compensates for the lack of detail authenticity. The form of combining the two loss functions is

(16) $$L_{total}=\lambda_{mse}L_{MSE}+\lambda_{adv}L_{adv}$$

where $\lambda_{mse}$ and $\lambda_{adv}$ are dynamic weight coefficients controlling the contributions of the two objective functions. Since the optimal trade-off between pixel-level precision and perceptual quality shifts fundamentally during the training process, relying on traditional fixed hyperparameters often leads to suboptimal convergence or early mode collapse. To address this, we translate the aforementioned trade-off analysis into a mathematical dynamic scheduling mechanism. Specifically, the coefficients are adaptively adjusted across different training stages using a multi-stage warm-up and linear scaling mechanism, tightly coupled with a cosine annealing learning rate strategy. The complete scheduling mechanism is defined as follows:

(17) $$\lambda_{mse}\left(t\right)=1$$

Here, the weight for the Mean Squared Error (MSE) loss is strictly maintained at 1 throughout the entire training process. This constant dominant value ensures that the network consistently receives stable gradient penalties for pixel-level structural differences, anchoring the fundamental anatomical topology of the reconstructed medical images and preventing spatial distortion:

(18) $$\lambda_{adv}\left(t\right)=\left\{\begin{array}{cc} 0 & 0\leq t<T_{warm}\\ \gamma \cdot \frac{t-T_{warm}}{T_{total}-T_{warm}} & T_{warm}\leq t\leq T_{total} \end{array}\right.$$

In this piecewise function, t denotes the current training epoch, and $T_{total}$ is the total number of epochs. $T_{warm}$ represents the structural warm-up period, which is set to 30% of $T_{total}$. During the warm-up phase, the adversarial weight is deactivated (set to 0) to force the generator to prioritize structural learning. Afterwards, it scales linearly to a maximum threshold defined by the hyperparameter γ, which is empirically set to 10^−3^. This controlled progressive scaling allows the model to synthesize natural high-frequency textures without destroying the stabilized underlying clinical structures:

(19) $$\eta \left(t\right)=\eta_{min}+\frac{1}{2}\left(\eta_{max}-\eta_{min}\right)\left(1+\cos\left(\pi \frac{t}{T_{total}}\right)\right)$$

The proposed algorithm synchronously controls the learning rate $\eta \left(t\right)$ via a cosine annealing strategy. The terms $\eta_{max}$ and $\eta_{min}$ represent the initial maximum learning rate and the terminal minimum learning rate, respectively. Smoothly decaying the learning rate allows the model to take large exploratory steps during the MSE-dominated early stages and perform fine-grained parameter updates during the highly sensitive adversarial fine-tuning stage, thereby stabilizing the overall convergence of the generative adversarial network.

**Figure 5:**
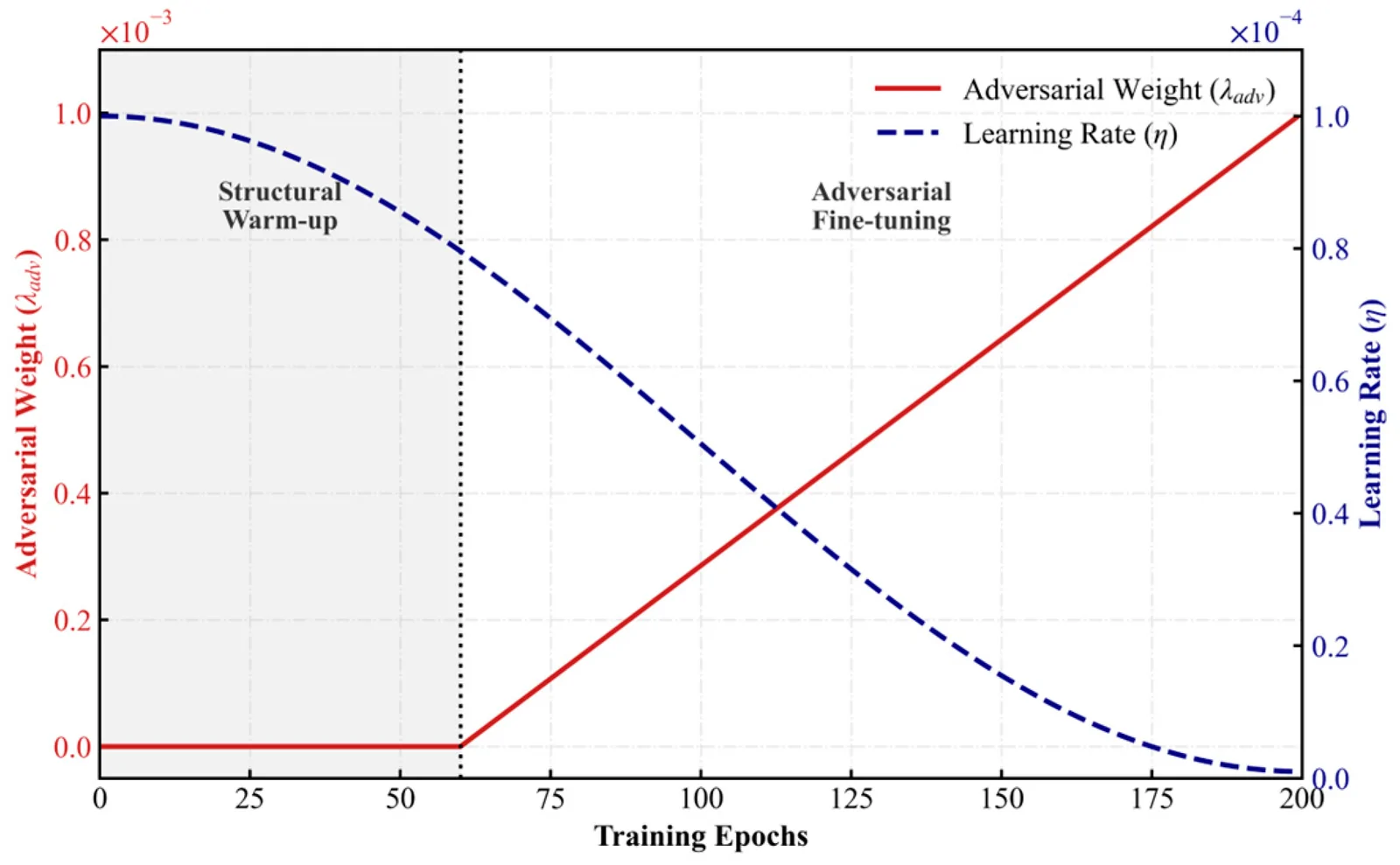
Dynamic scheduling trajectory of adversarial weight and learning rate during training.

To intuitively demonstrate the proposed dynamic scheduling mechanism, the exact trajectories of the adversarial weight $\lambda_{adv}$ and the learning rate $\eta \left(t\right)$ across the training epochs are visualized in Figure 5.

As illustrated, the training process is explicitly divided into two distinct phases. During the initial structural warm-up phase, the generative adversarial network operates exclusively under the MSE loss penalty to establish a robust anatomical baseline. Once the training enters the adversarial fine-tuning phase, the progressive linear introduction of the adversarial weight, coupled with the smooth cosine decay of the learning rate, synergistically prevents gradient explosion and early mode collapse. This precisely controlled training dynamic ensures that high-frequency clinical textures are synthesized without distorting the underlying macroscopic bone structures.

## 3. Experiments

### 3.1. Experimental Environment and Parameter Settings

This experiment was conducted on the Windows 10 operating system (Microsoft, Redmond, WA, USA). The Python-based PyCharm Community Edition 2023.3.6 (JetBrains, Prague, Czech Republic) was utilized as the simulation software alongside the PyTorch 2.3.1 deep learning framework with CUDA 11.8 (NVIDIA, Santa Clara, CA, USA), with Python 3.9 (Python Software Foundation, Wilmington, DE, USA) serving as the programming language version.. During the training process, the parameters of both the generator and discriminator were optimized using the Adam optimization algorithm [28], with momentum parameters set to $\beta_{1}=0.9$, $\beta_{2}=0.99$, and $\epsilon =10^{-8}$. The model was trained for a total of 200 epochs with a batch size of 16. The base learning rate for the generator was governed by a cosine annealing strategy, decaying smoothly from an initial maximum learning rate of 0.0001 to a terminal minimum learning rate of $10^{-6}$. Simultaneously, the discriminator utilized a fixed learning rate of $5\times 10^{-5}$ and a generator-to-discriminator update ratio of 1:1 to maintain a balanced adversarial game. The utilized hardware parameters are presented in Table 1.

### 3.2. Experimental Datasets

**Figure 6:**
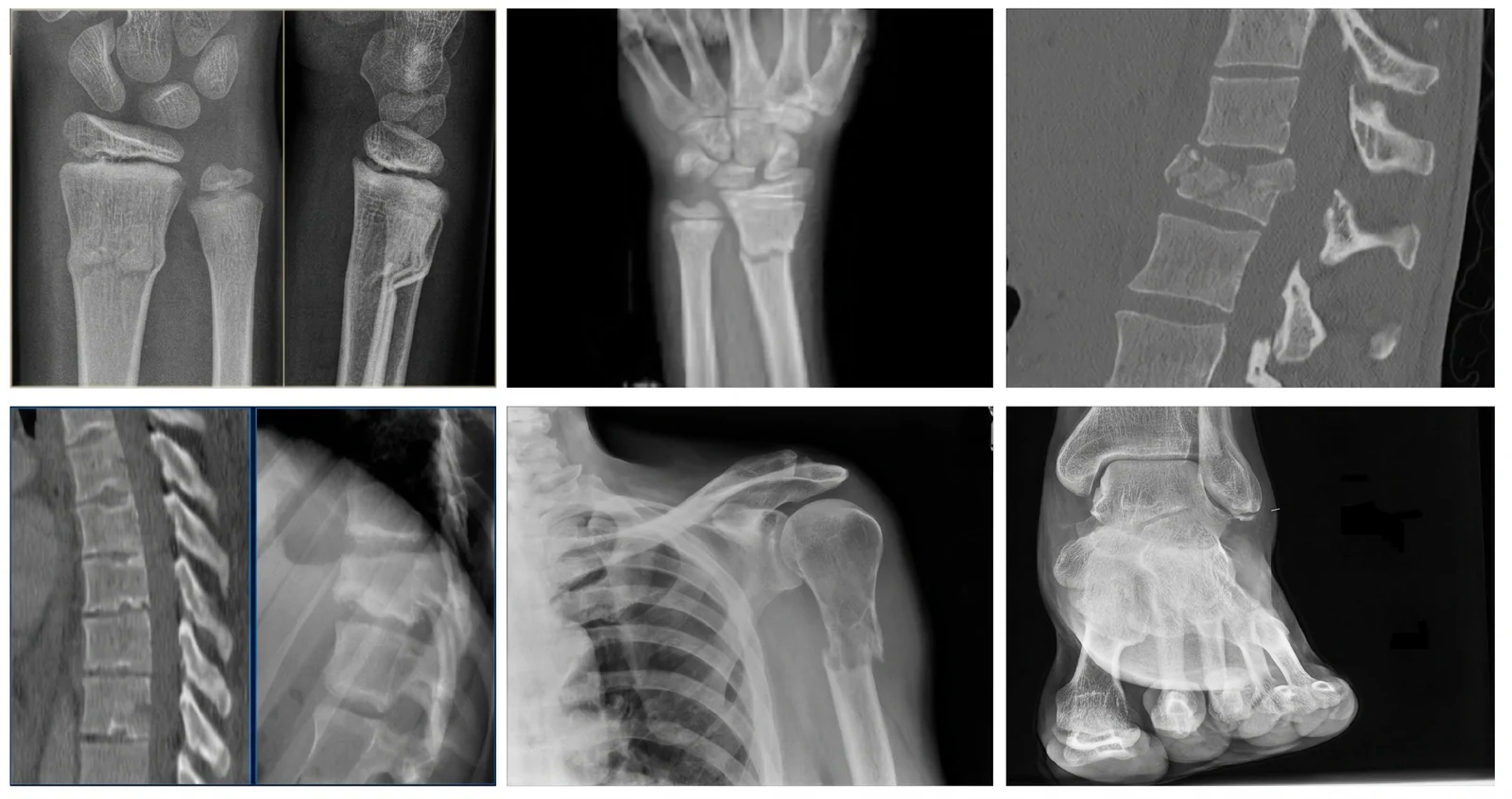
Bone Break Classifier Dataset.

In this paper, the public Bone Break Classifier Dataset is used as the main training set, which consists of 1750 images as shown in Figure 6. And the DIV2K dataset [29], which contains 800 high-resolution images in 2K HD, is also used. In order to evaluate the effectiveness of the algorithm, this paper will evaluate on five benchmark datasets: Set5 [30], Set14, BSD100 [31], Urban100 [32], and the publicly available medical bone dataset Bone FD (Bone Fracture Detection: Computer Vision Project).

### 3.3. Experimental Evaluation Index

To comprehensively evaluate the performance of the proposed super-resolution model, this study employs a combination of distortion-based and perception-based metrics. While traditional pixel-wise metrics objectively quantify numerical deviations, deep-feature-based metrics are introduced to better align with human visual perception and clinical diagnostic requirements.

The Peak Signal-to-Noise Ratio (PSNR) is a standard objective metric used to measure the pixel-wise error between a reference image x and a reconstructed image y. It is defined as

(20) $$PSNR=10\cdot \log_{10}\left(\frac{MAX^{2}}{MSE\left(x,y\right)}\right)$$

where MAX represents the maximum possible pixel value of the image (e.g., 255 for 8-bit images), and MSE denotes the Mean Squared Error between x and y. A higher PSNR indicates lower pixel-level distortion.

Structural Similarity (SSIM) evaluates image fidelity based on the characteristics of the human visual system by modeling luminance, contrast, and structural information. For two images x and y, SSIM is formulated as

(21) $$SSIM\left(x,y\right)=\frac{\left(2\mu_{x}\mu_{y}+C_{1}\right)\left(2\sigma_{xy}+C_{2}\right)}{\left(\mu_{x}^{2}+\mu_{y}^{2}+C_{1}\right)\left(\sigma_{x}^{2}+\sigma_{y}^{2}+C_{2}\right)}$$

where $\mu_{x}$ and $\mu_{y}$ denote the mean intensities, $\sigma_{x}^{2}$ and $\sigma_{y}^{2}$ are the variances, $\sigma_{xy}$ is the covariance, and $C_{1}$ and $C_{2}$ are small constants to ensure numerical stability. The SSIM value ranges from $\left[-1,1\right]$, with a value closer to 1 implying higher structural fidelity.

However, models purely optimized for pixel-wise metrics often yield overly smooth results. To evaluate the naturalness of high-frequency textures, two deep-feature perceptual metrics are additionally introduced.

Learned Perceptual Image Patch Similarity (LPIPS) computes the weighted $L_{2}$ distance of feature activations extracted from a pre-trained deep network (e.g., VGG). Assuming ${\hat{x}}^{l}$ and ${\hat{y}}^{l}$ represent the normalized feature maps extracted at layer l for images x and y, LPIPS is formulated as

(22) $$LPIPS\left(x,y\right)=\sum_{l}\frac{1}{H_{l}W_{l}}\sum_{h,w}||w_{l}⊙\left({\hat{x}}_{hw}^{l}-{\hat{y}}_{hw}^{l}\right){\left|\right|}_{2}^{2}$$

where $H_{l}$ and $W_{l}$ are the spatial dimensions of the layer l, $w_{l}$ is the channel-wise scaling vector, and ⊙ denotes element-wise multiplication. A lower LPIPS score indicates higher perceptual similarity. To ensure rigorous and reproducible perceptual evaluation, the exact LPIPS configuration used in our experiments is detailed as follows: The metric was computed utilizing the lpips package version 0.1.4 equipped with an AlexNet backbone. Linear calibrated weights from version 0.1 were explicitly activated. Prior to feature extraction, all input image tensors were strictly normalized from the original 0 to 1 range into a −1 to 1 range. For single-channel medical images such as Bone FD, the inputs were expanded along the channel dimension by repeating the Y channel three times to construct a pseudo-RGB representation, which aligns perfectly with the expected input dimensions of the pre-trained backbone.

### 3.4. Objective Comparison and Analysis of Different Algorithms

**Figure 7:**
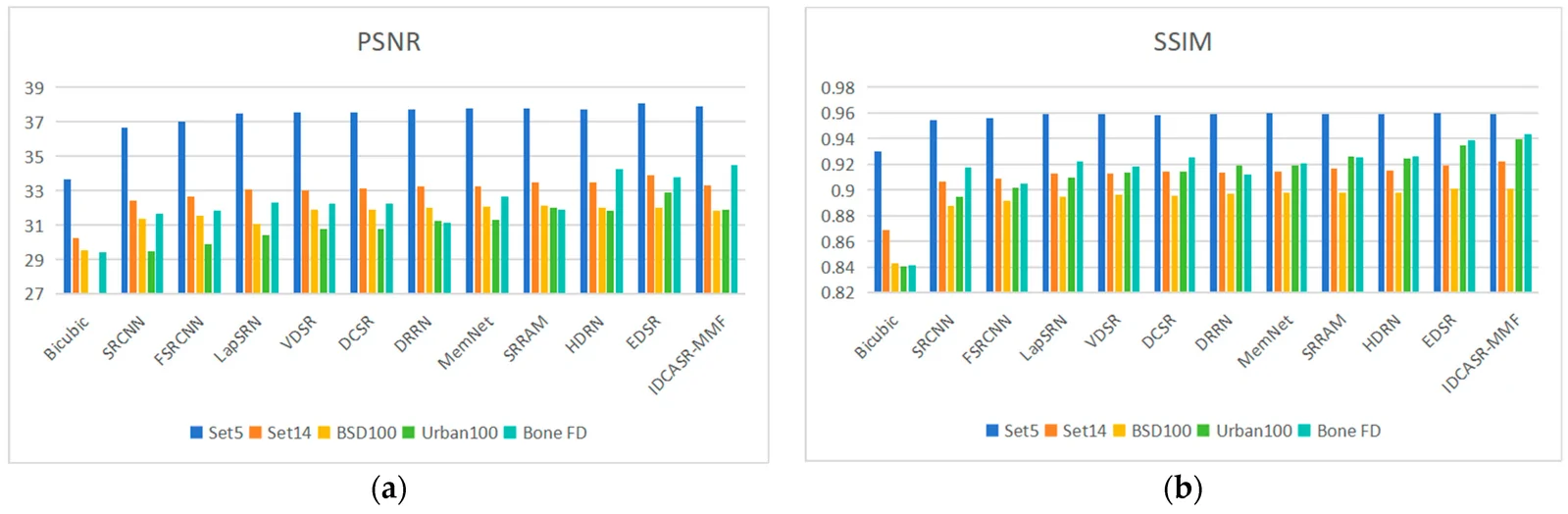
Comparison of 12 algorithms with an amplification factor of 2: (**a**) PSNR; (**b**) SSIM.

**Figure 8:**
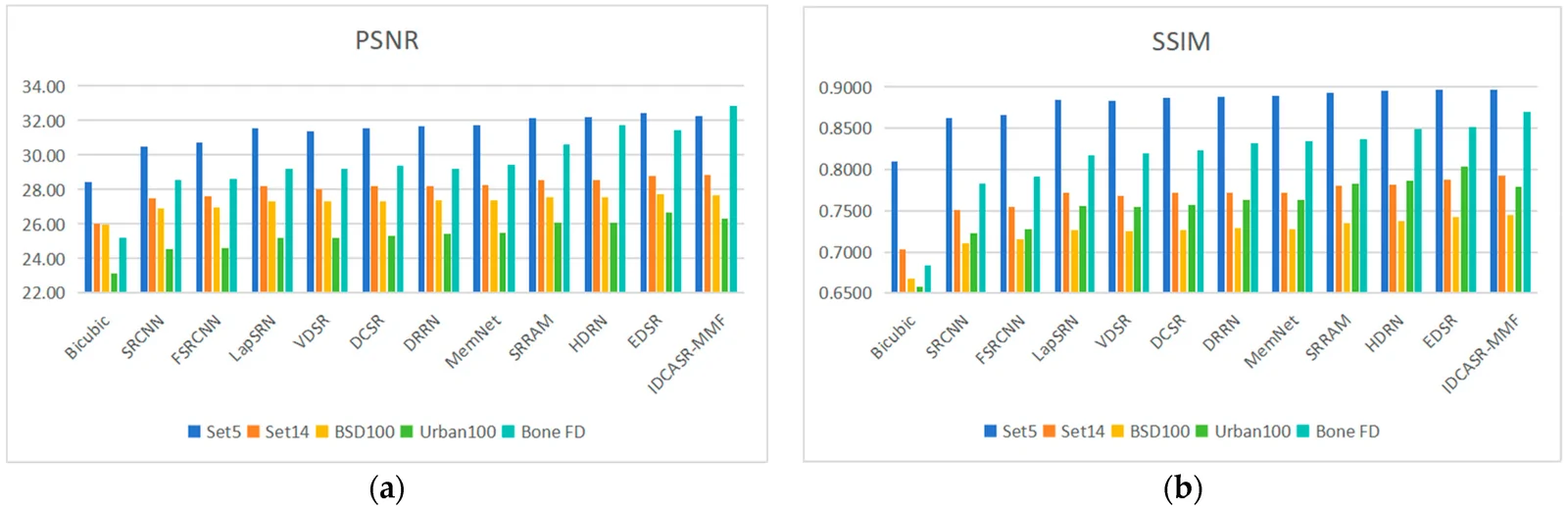
Comparison of 12 algorithms with an amplification factor of 4: (**a**) PSNR; (**b**) SSIM.

In order to evaluate the performance of the proposed algorithm, 11 mainstream algorithms are selected as comparison algorithms: Bicubic, SRCNN, FSRCNN [33], LapSRN [34], VDSR, DCSR, DRRN [35], MemNet [36], SRRAM [37], HDRN [38], and EDSR. The experimental comparison data of the algorithms in this paper, with 11 mainstream algorithms in the case of amplification factors of 2 and 4 for Set5, Set14, BSD100, Urban100 and the public medical bone dataset Bone FD test set, are shown in Table 2 and Table 3, and the data histogram is shown in Figure 7 and Figure 8, and we can see Set5, Set14, BSD100, Urban100 and the public medical skeleton dataset under the amplification factors of 2 and 4, respectively. Compared with the 11 mainstream algorithms, the optimal and suboptimal PSNR and SSIM are obtained in most of the general test datasets of the proposed algorithm. In the medical image test dataset, both optimal and sub-optimal PSNR and SSIM were obtained.

At magnification of 2, the tabular results show that EDSR is slightly dominant on the Set5 dataset, with a PSNR of 38.11 and an IDCASR-MMF of 37.92, a difference of 0.19. However, in the same scenario, the SSIM of IDCASR-MMF is only 0.0014 lower than EDSR, and the visual consistency remains at a high level. In addition, on Urban100, EDSR’s PSNR is about 32.93, while IDCASR-MMF is 31.91, a difference of 1.02, but IDCASR-MMF’s SSIM is significantly higher than EDSR’s, showing that it has stronger fidelity for structure and detail in urban scenes. The PSNR of 0.74 and the SSIM of 0.0053 were higher than those of EDSR on the Bone FD dataset, respectively, indicating that IDCASR-MMF was superior in the restoration of key parts such as bone details.

When the magnification is 4, the improvement of IDCASR-MMF on Bone FD is the most prominent: its PSNR can reach 32.87 and SSIM is 0.8702, while the EDSR is 31.43 and 0.8516 under the same conditions, respectively, with a difference of 1.44 and 0.0186, which further indicates that the proposed method has a stronger ability to restore the details of medical images at high magnification. In contrast, on Set5, the PSNR and SSIM of EDSR are slightly higher than IDCASR-MMF, with a difference of only 0.2 and 0.0004, indicating that the performances of the two are very similar in general-purpose image scenarios. For Urban100, the PSNR of EDSR is 0.30 higher than IDCASR-MMF, and the SSIM is about 0.0246 higher, indicating that it still has certain advantages in urban structural texture, but IDCASR-MMF shows more significant detail fidelity and structural recovery capabilities in other datasets, especially medical images.

While Table 3 demonstrates the high reconstruction fidelity of our method in terms of pixel-wise metrics, these standard criteria often fail to reflect the synthesized textural naturalness under larger scaling factors. To establish a more comprehensive evaluation regarding human visual perception, we further report the LPIPS results under the challenging 4× scale factor in Table 4.

As presented in Table 4, evaluating perceptual quality in medical image super-resolution necessitates an objective analysis of the perception–distortion trade-off, which is essential for clinical diagnosis. While traditional models optimized solely by Mean Squared Error (MSE) yield high pixel-wise metrics, they tend to generate over-smoothed textures that can obscure high-frequency details, such as microcalcifications and subtle lesions. The proposed IDCASR-MMF mitigates this issue by integrating a dynamic mixed-loss scheduling mechanism with the DCSE module. Rather than producing an isolated perceptual deviation, IDCASR-MMF improves LPIPS scores by targeting clinically relevant high-frequency pathological structures during the adversarial fine-tuning phase. This mechanism allows the model to reconstruct fine micro-pathological features, such as bone trabeculae, alongside distinct tissue boundaries. Consequently, IDCASR-MMF establishes an effective balance between macroscopic anatomical fidelity and microscopic perceptual quality, offering visual support for downstream diagnostic tasks and precision medicine.

**Figure 9:**
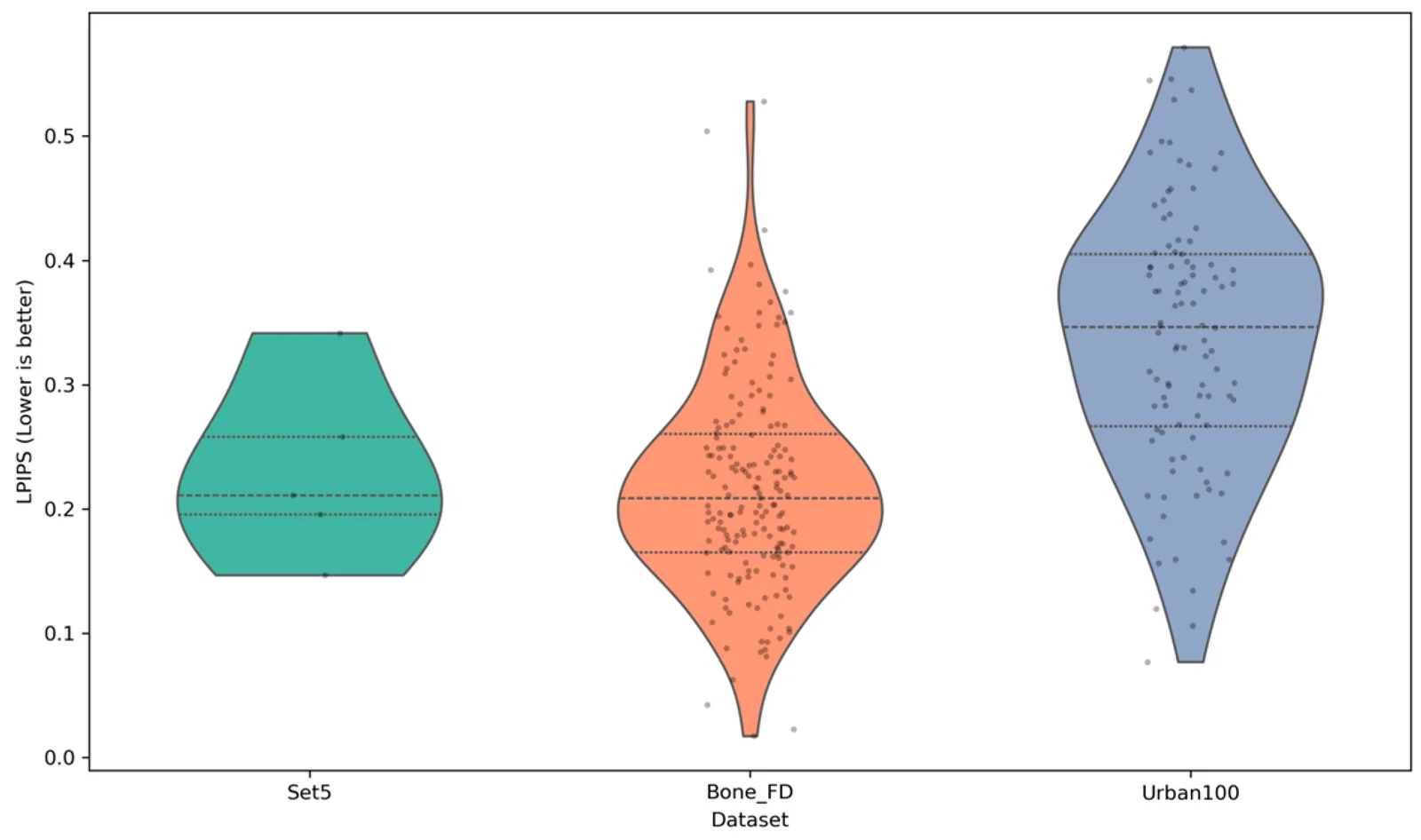
Violin plots of per-image LPIPS on Set5, Bone_FD, and Urban100 datasets (dashed lines inside the violins denote the 25th, median, and 75th percentiles).

To provide a comprehensive assessment beyond dataset means and analyze perceptual variance, Figure 9 illustrates the per-image LPIPS distributions via violin plots for the Set5, Bone FD, and Urban100 datasets using the IDCASR-MMF model. The distribution characteristics reveal that the model yields lower LPIPS values for the majority of samples, which are densely clustered in the lower quartile range. Notably, while the model exhibits a slight performance decrease on the Urban100 dataset, it demonstrates distinct improvements on the Bone FD medical dataset. By reporting the mean alongside the standard deviation in Table 4 and visualizing the per-image distributions, the statistical variance associated with the GAN-based synthesis is objectively measured. These findings indicate that the perceptual improvements are consistent across most samples, offering a clinically relevant evaluation of the model’s performance.

### 3.5. Subjective Evaluation Methods of Different Algorithms

In order to further verify the effectiveness of the proposed algorithm, we carried out a comparative experiment on the subjective reconstruction effect. In the experiment, the local area with complete structural information in the image was enlarged and fused and compared on the right side of the reference image, in which five algorithms with better data in the objective evaluation experiment were selected for comparison, namely LapSRN, VDSR, SRRAM, HDRN, and EDSR, and the visual effect of the reconstructed SR image is shown in the figure.

**Figure 10:**
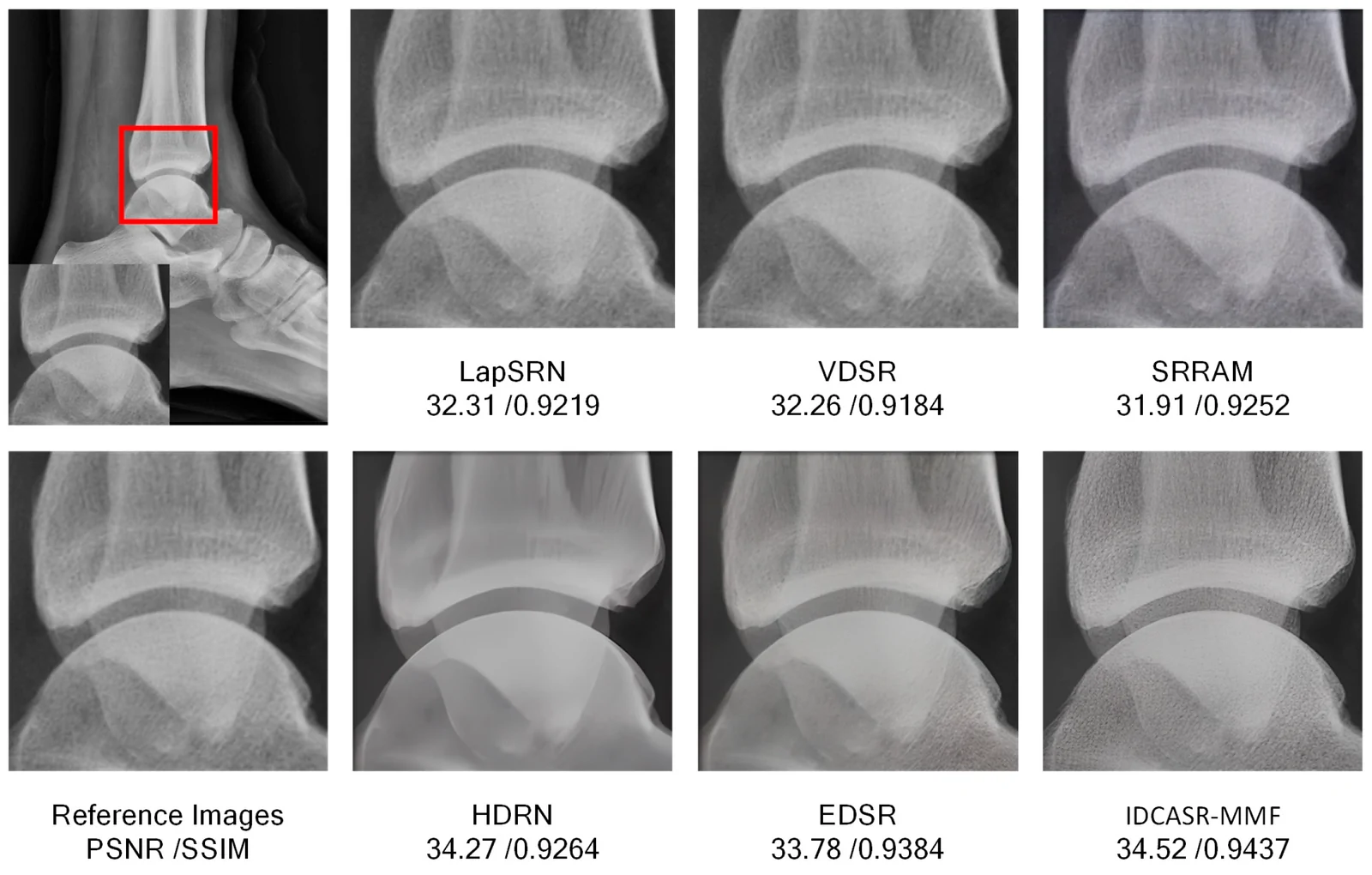
Visual comparison of reconstruction effects of different algorithms at a magnification factor of 2.

**Figure 11:**
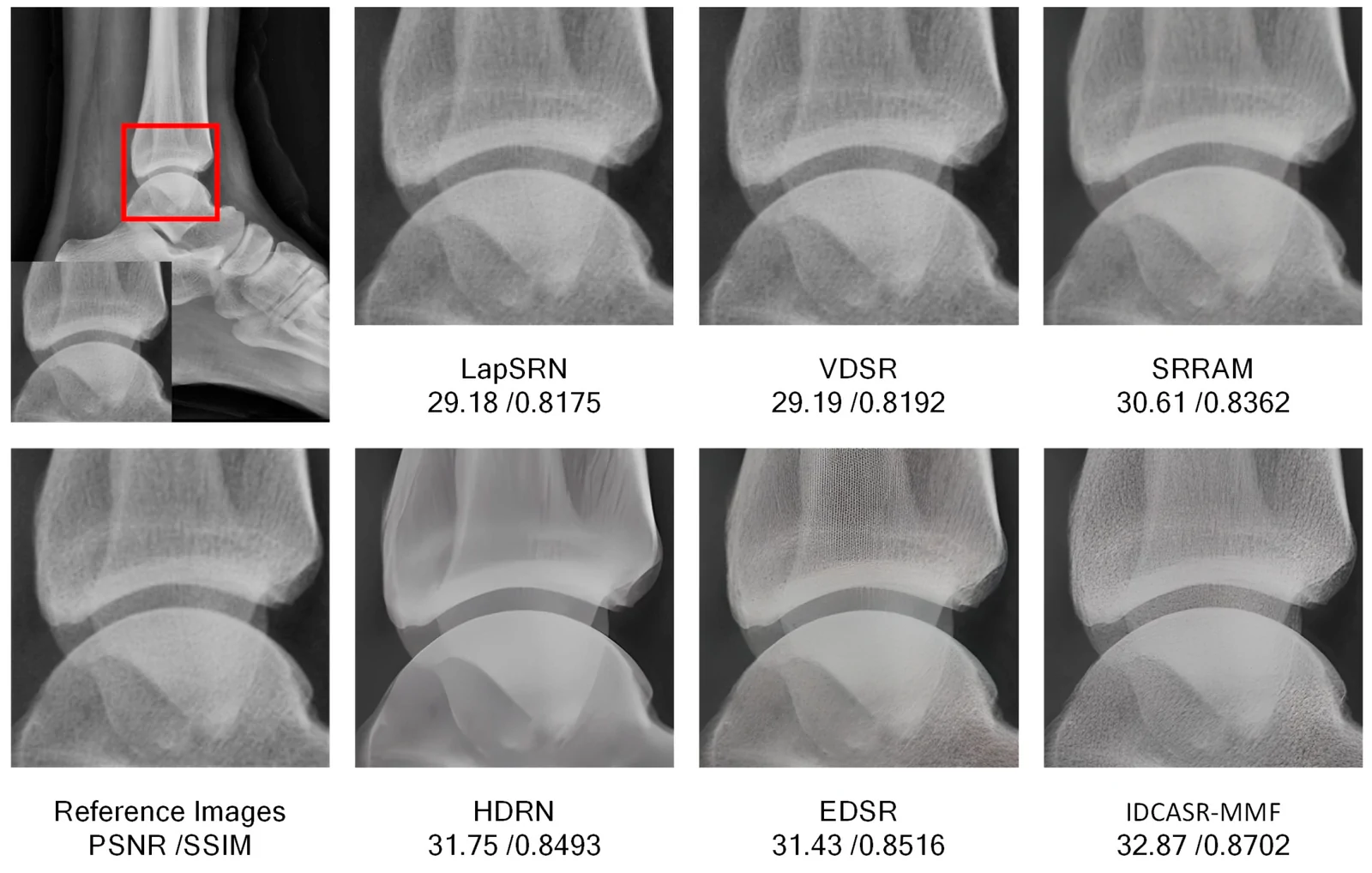
Visual comparison of reconstruction effects of different algorithms at a magnification factor of 4.

It can be observed from Figure 10 and Figure 11 that when the magnification factors are 2 and 4, although the LapSRN and VDSR have obtained relatively ideal values in the objective indicators, the reconstructed images generated by them are still slightly blurry and lack sufficient detail compared with the original images. SRRAM has a noticeable color cast visually; Although HDRN has a strong sense of structure, it is almost lost due to excessive smoothing, which is difficult to meet the detailed needs of medical images. Compared with the above method, EDSR has been significantly improved, and the structural edges of the output image are clearer, and the detailed texture is richer, but a small number of mesh textures that do not belong to the bone itself are still produced, and the restoration accuracy of some bone structures needs to be improved. IDCASR-MMF shows more significant details in skeletal images, restores more high-frequency information carried by images, and has obvious advantages over other algorithms.

Based on the above comparative experiments, compared with the mainstream algorithms and the traditional CNN super-resolution reconstruction algorithms, the proposed algorithm not only achieves a great performance improvement in PSNR value and SSIM value but also has better visual effect in the details of the reconstructed image. In the field of medical imaging, the algorithm in this paper has been significantly improved compared with the mainstream algorithms, as the algorithm can capture multi-scale information in different regions, focus on high-frequency components, and extract multi-level contextual information in a variety of different regions so that they can compensate each other to obtain better high-frequency information. Moreover, the reconstructed medical image is clearer in texture details, and the overall visual effect is better, and contains more original image information of medical images.

### 3.6. Ablation Experiments

After comparing with other mainstream algorithms, we further designed ablation experiments to analyze the contribution of each key module of IDCASR-MMF to the model performance. Specifically, we remove or replace different components of the model in turn, construct some simplified versions, and compare them in the same training and testing environments. Through the evaluation of objective indicators, the importance of each module in the super-resolution reconstruction process can be more intuitively verified.

#### 3.6.1. Ablation Settings

For illustrative purposes, the full model is denoted as “IDCASR-MMF”. Focusing on the three core improvement points of the model, we remove or retain only some components in turn, and construct the following experimental groups as summarized in Table 5:

#### 3.6.2. Experimental Results and Analysis

**Figure 12:**
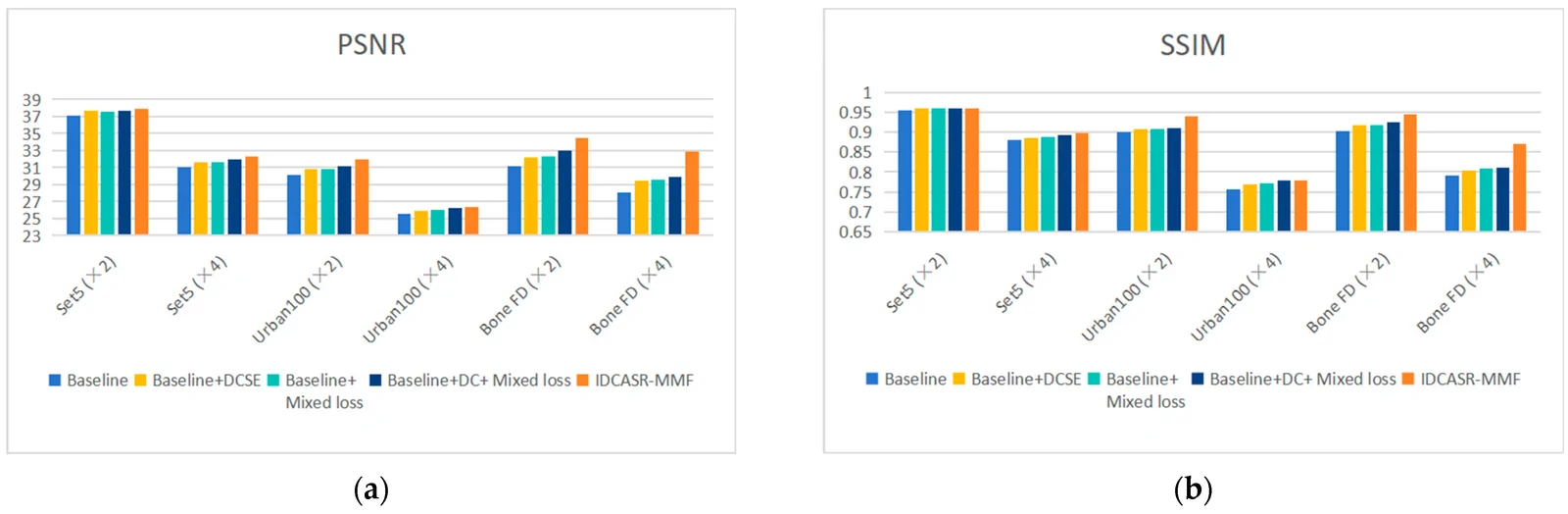
Comparison of the utility of each module of the ablation experiment: (**a**) PSNR; (**b**) SSIM.

Table 6 and Figure 12 show the average PSNR/SSIM comparison for various ablation settings at Set5, Urban100, and Bone FD at magnification factors 2 and 4 (the results of the other datasets have a similar trend and will not be listed here due to space limitations). Values in bold represent the best result in the row, and underscores indicate the next best result.

As can be seen from Table 6 and Figure 12, each key module has different degrees of gain in model performance, and the full model (IDCASR-MMF) performs best in all scenarios. Taking the Bone FD dataset as an example, when the amplification factor is ×4, the PSNR of the baseline (no DCSE/MSE only) scheme is only 28.10, while the IDCASR-MMF of the full model reaches 32.87, a gap of 4.77. SSIM also increased from 0.7923 to 0.8702, an increase of more than 0.0779. For example, on the Set5 dataset, when the amplification factor is ×2, the PSNR of Baseline is 37.10, and the IDCASR-MMF is increased to 37.92, although the increase is slightly smaller, but it still proves the effectiveness of the dilated convolutional attention module and the mixed loss function. In particular, when comparing “Baseline + DC (de-SE) + mixed loss” and “Baseline + DC + mixed loss”, the PSNR of the former can reach 33.02 in the Bone FD ×2 scenario, while the latter is further improved to 34.52, indicating that the introduction of SE channel attention can further enhance the model’s ability to capture detailed features on the basis of the existing dilated convolution and mixed loss.

The comparison between Baseline and Baseline + DC shows that PSNR and SSIM can be significantly improved by introducing only the dilated convolution and channel attention modules, indicating that the large receptive field and channel attention of the dilated convolution are indeed effective in enhancing the detail texture. Baseline + Mixed Loss (MSE + Adversarial) also achieved better gain than Baseline, demonstrating that adversarial loss helps the generator learn more realistic texture details and outperforms a single MSE in perceived quality. Furthermore, the stable convergence of this mixed loss heavily relies on the proposed dynamic scheduling mechanism. By prioritizing the MSE loss during the warm-up phase and linearly scaling the adversarial penalty later, the model successfully avoids the structural distortion and grid artifacts that typically occur when static adversarial weights are applied directly to medical images.

When the dilated convolution (excluding SE) is combined with the mixed loss (Baseline + DC + mixed loss), the results are further improved, and the synergistic effect of the dilated convolution and the adversarial loss is realized. Dilated convolution provides richer multi-scale context information for the model, and adversarial loss plays a key role in high-frequency texture reconstruction. Finally, the complete model (IDCASR-MMF) performed best among all comparison groups, indicating that SE channel attention and dilated convolution are complementary, and combined with the mixing loss, the pixel accuracy and perceptual quality can be optimized at the same time. Overall, the modules complement each other, ultimately helping IDCASR-MMF achieve better and more stable reconstruction performance on both medical images and general datasets. Especially on the medical dataset (Bone FD), the fidelity and sharpness of bone details are significantly improved by IDCASR-MMF.

Based on the above ablation experiments, the multi-branch and multi-scale design of dilated convolution and attention mechanism (DCSE) can significantly improve the reconstruction effect of bone and other fine structures, and the hybrid loss function achieves a better balance between pixel-level accuracy and perceptual quality. The complete IDCASR-MMF model performed well in both subjective and objective evaluations, which provided a feasible idea for the subsequent expansion to more medical imaging scenarios.

### 3.7. Cross-Dataset Generalization Evaluation

To evaluate the algorithm’s generalization capability across unseen data distributions, a zero-shot cross-dataset evaluation was conducted using the CT-KIDNEY-DATASET-Normal-Cyst-Tumor-Stone. The tissue texture and background complexity of this dataset are higher than those of the original bone dataset. During inference, the IDCASR-MMF weights trained exclusively on the bone dataset were applied directly without fine-tuning.

In the quantitative evaluation, all models experienced performance degradation due to the domain shift. Specifically, the baseline EDSR exhibited a PSNR drop of 0.58 dB and an SSIM drop of 0.0094. The proposed IDCASR-MMF experienced a PSNR decrease of 0.92 dB and an SSIM drop of 0.0088. The larger PSNR variation in IDCASR-MMF is theoretically consistent with the characteristics of adversarial-driven models, which generate high-frequency details on unseen soft-tissue distributions, thereby increasing pixel-wise mean squared errors. However, the structural degradation of IDCASR-MMF was lower than that of EDSR. This indicates that the dynamic scheduling mechanism maintains structural preservation capabilities across different data distributions, yielding an overall performance of 31.95 dB for PSNR and 0.8614 for SSIM.

**Figure 13:**
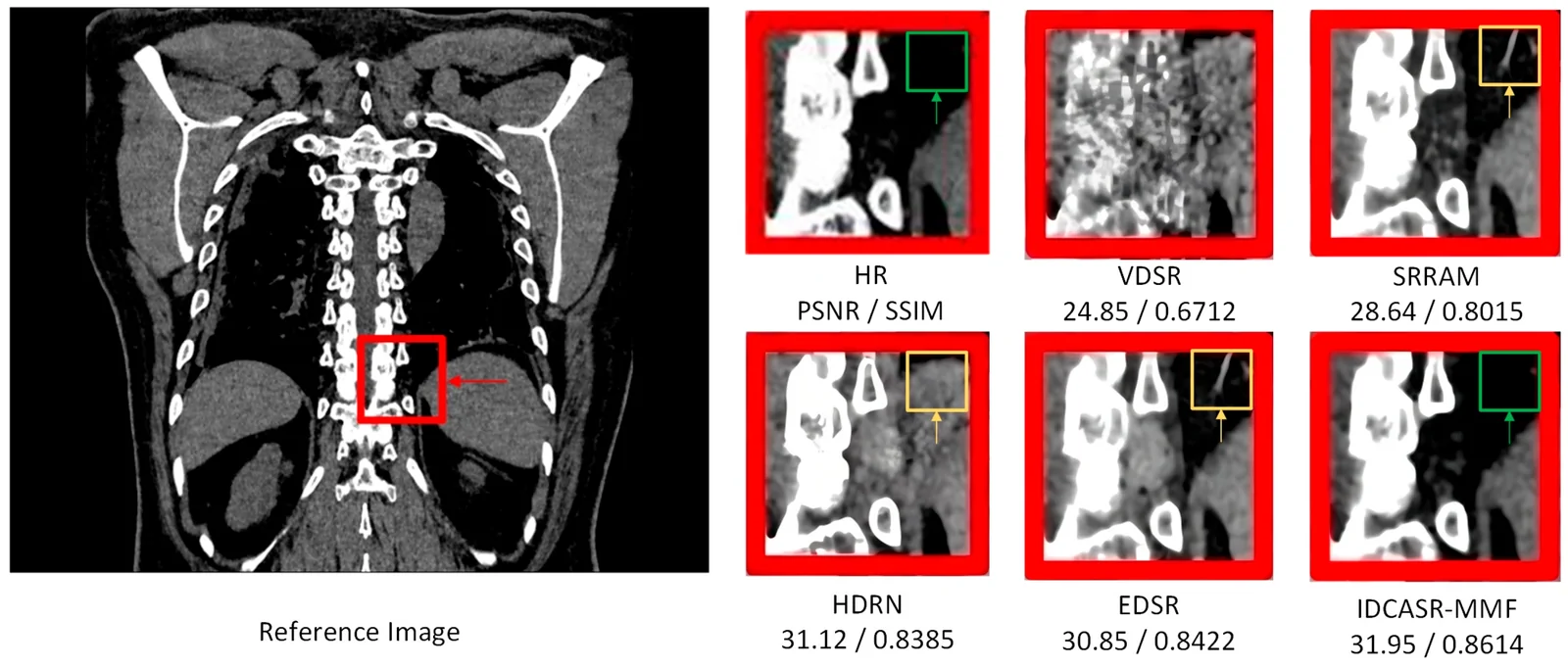
Visual comparison of reconstruction artifacts in the cross-dataset evaluation.

In the qualitative evaluation, Figure 13 utilizes colored bounding boxes to display artifact generation and suppression at a 4× magnification factor. Due to the increased structural complexity, baseline models exhibit limitations in maintaining anatomical fidelity. Specifically, SRRAM, HDRN, and EDSR generate streak-like artifacts and structural hallucinations in soft tissue regions, indicated by yellow boxes. The VDSR model introduces pixel-level noise. In contrast, the high-resolution reference image and IDCASR-MMF present anatomical backgrounds without the aforementioned artifacts, indicated by green boxes. This observation demonstrates that IDCASR-MMF mitigates structural hallucinations and preserves anatomical integrity when applied to complex medical imaging data outside the training domain.

### 3.8. Downstream Clinical Task Evaluation

Image quality metrics, such as peak signal-to-noise ratio PSNR and structural similarity SSIM, evaluate pixel-wise distortions but do not fully reflect the preservation of critical pathological features required for clinical diagnosis. To assess the recognition probability of medical conditions on the reconstructed images, a downstream bone fracture classification task is introduced.

This experiment employs a standard residual network ResNet-18 to perform binary classification inference on the Bone Break Classifier Dataset. The original high-resolution HR images are evaluated to establish the performance upper bound. Subsequently, the low-resolution images upsampled by a factor of 4 using Bicubic interpolation, the baseline model EDSR, and the proposed IDCASR-MMF method are fed into the same classification pipeline. The evaluation metrics include Accuracy, Precision, Recall, and the area under the receiver operating characteristic curve (AUC).

As presented in Table 7, the classification performance of images reconstructed by IDCASR-MMF outperforms the other comparative methods. Although the EDSR model demonstrates high objective metrics in the structural evaluation, its optimization mechanism driven by mean squared error MSE leads to the smoothing of micro-fracture lines, resulting in a classification accuracy of 84.50%. In contrast, IDCASR-MMF preserves high-frequency features with clinical diagnostic value through dynamic mixed-loss scheduling. The accuracy reaches 89.85% and the AUC reaches 0.928, which approach the performance upper bound of the HR images. This evaluation indicates that the proposed algorithm improves the performance of computer-aided diagnosis in clinical scenarios with restricted hardware conditions.

## 4. Conclusions

In this paper, we propose a novel medical image super-resolution reconstruction algorithm, IDCASR-MMF, aimed at resolving the inherent contradiction between high-frequency detail restoration and macro-structural preservation in clinical imaging. The main contributions and conclusions of this study are summarized as follows.

We developed a multi-module fusion network that integrates multi-scale dilated convolutions and channel attention mechanisms. This architectural design effectively expands the receptive field with minimal computational overhead, empowering the model to accurately capture and dynamically calibrate micro-pathological features.

A dynamic scheduling mechanism incorporating multi-stage warm-up and cosine annealing is designed to synergize the Mean Squared Error loss and adversarial losses. This specific training trajectory successfully balances pixel-level anatomical accuracy with high-frequency texture recovery, effectively overcoming the over-smoothing issue of traditional MSE while avoiding GAN-induced structural hallucinations.

We demonstrated the clinical reliability of IDCASR-MMF through extensive quantitative and qualitative evaluations on the target Bone FD dataset and general benchmarks. The algorithm achieves an optimal perception–distortion tradeoff by significantly improving perceptual metrics while maintaining strict pixel-level structural fidelity, proving its robustness for downstream diagnostic tasks.

Future research will focus on developing cross-modal adaptive mechanisms, aiming to seamlessly transfer this domain-specific architectural advantage to other medical modalities, such as MRI and Ultrasound, to establish a unified multi-modal medical image enhancement framework.

## Figures and Tables

**Table 1:** Experimental Hardware Platform.

Hardware	Specifications
CPU	12th Gen Intel(R) Core(TM) i5-12400F Intel, Santa Clara, CA, USA
GPU	NVIDIA GeForce RTX 4060 Ti NVIDIA, Santa Clara, CA, USA
Memory	32 GB KingBank, Shenzhen, China

**Table 2:** Comparison of PSNR and SSIM of 12 Algorithms on 5 Test Sets When the Magnification Factor Is 2.

Algorithm	Set5		Set14		BSD100		Urban100		Bone FD	
	PSNR	SSIM	PSNR	SSIM	PSNR	SSIM	PSNR	SSIM	PSNR	SSIM
Bicubic	33.66	0.9299	30.24	0.8688	29.56	0.8431	26.88	0.8403	29.41	0.8417
SRCNN	36.66	0.9542	32.45	0.9067	31.36	0.8879	29.5	0.8946	31.64	0.9173
FSRCNN	37.05	0.9560	32.66	0.9090	31.53	0.8920	29.88	0.9020	31.85	0.9053
LapSRN	37.52	0.9591	33.08	0.9130	31.08	0.8950	30.41	0.9101	32.31	0.9219
VDSR	37.53	0.9590	33.05	0.9130	31.90	0.8960	30.77	0.9140	32.26	0.9184
DCSR	37.54	0.9587	33.14	0.9141	31.90	0.8959	30.76	0.9142	32.28	0.9258
DRRN	37.74	0.9591	33.23	0.9136	32.05	0.8973	31.23	0.9188	31.15	0.9117
MemNet	37.78	0.9597	33.28	0.9142	32.08	0.8978	31.31	0.9195	32.68	0.9207
SRRAM	37.82	0.9592	33.48	0.9171	**32.12**	0.8983	32.05	0.9264	31.91	0.9252
HDRN	37.75	0.9590	33.49	0.9150	32.03	0.8980	31.87	0.9250	34.27	0.9264
EDSR	**38.11**	**0.9602**	**33.92**	0.9195	32.02	**0.9013**	**32.93**	0.9251	33.78	0.9384
IDCASR-MMF	37.92	0.9588	33.34	**0.9219**	31.87	0.9012	31.91	**0.9394**	**34.52**	**0.9437**

Note: The bold values indicate the best results, and the underlined values indicate the suboptimal results.

**Table 3:** Comparison of PSNR and SSIM for 12 Algorithms on 5 Test Sets with an Amplification Factor of 4.

Algorithm	Set5		Set14		BSD100		Urban100		Bone FD	
	PSNR	SSIM	PSNR	SSIM	PSNR	SSIM	PSNR	SSIM	PSNR	SSIM
Bicubic	28.42	0.8104	26.00	0.7027	25.96	0.6675	23.14	0.6577	25.19	0.6836
SRCNN	30.48	0.8628	27.50	0.7513	26.90	0.7101	24.52	0.7221	28.58	0.7824
FSRCNN	30.72	0.8660	27.61	0.7550	26.98	0.7150	24.62	0.7280	28.64	0.7911
LapSRN	31.54	0.8850	28.19	0.7720	27.32	0.7270	25.21	0.7560	29.18	0.8175
VDSR	31.35	0.8830	28.02	0.7680	27.29	0.7251	25.18	0.7540	29.19	0.8192
DCSR	31.58	0.8870	28.21	0.7715	27.32	0.7264	25.30	0.7575	29.35	0.8228
DRRN	31.68	0.8888	28.21	0.7721	27.38	0.7284	25.44	0.7638	29.18	0.8314
MemNet	31.74	0.8893	28.26	0.7723	27.40	0.7281	25.50	0.7630	29.42	0.8342
SRRAM	32.13	0.8932	28.54	0.7800	27.56	0.7350	**26.65**	0.7834	30.61	0.8362
HDRN	32.23	0.8960	28.58	0.7810	27.53	0.7370	26.09	0.7870	31.75	0.8493
EDSR	**32.46**	**0.8968**	28.80	0.7876	**27.71**	0.7320	26.64	**0.8033**	31.43	0.8516
IDCASR-MMF	32.26	0.8964	**28.83**	**0.7926**	27.66	**0.7454**	26.34	0.7787	**32.87**	**0.8702**

Note: The bold values indicate the best results, and the underlined values indicate the suboptimal results.

**Table 4:** Comparison of LPIPS for 12 Algorithms on 5 Test Sets with an Amplification Factor of 4.

Algorithm	Set5	Set14	BSD100	Urban100	Bone FD
	LPIPS	LPIPS	LPIPS	LPIPS	LPIPS
Bicubic	0.4124	0.4573	0.4859	0.5112	0.4481
SRCNN	0.3031	0.3462	0.3687	0.3979	0.3276
FSRCNN	0.2948	0.3371	0.3606	0.3930	0.3193
LapSRN	0.2693	0.3053	0.3345	0.3668	0.2934
VDSR	0.2633	0.3013	0.3311	0.3589	0.2872
DCSR	0.2596	0.2964	0.3164	0.3494	0.2841
DRRN	0.2517	0.2918	0.3127	0.3426	0.2750
MemNet	0.2435	0.2855	0.3111	0.3392	0.2676
SRRAM	0.2297	0.2674	0.2894	0.3232	0.2515
HDRN	0.2267	0.2644	0.2883	0.3210	0.2415
EDSR	0.2158	0.2512	0.2746	0.3061	0.2315
IDCASR-MMF	0.2108 ± 0.0658	0.2536 ± 0.1047	0.2882 ± 0.0769	0.3382 ± 0.1065	0.2171 ± 0.0832

**Table 5:** Ablation Experimental Groups.

Model Configuration	DC Module	SE Module	Mixed Loss Function
Baseline	⊠	⊠	⊠
Baseline + DCSE	☑	☑	⊠
Baseline + Mixed loss	⊠	⊠	☑
Baseline + DC+ Mixed loss	☑	⊠	☑
IDCASR-MMF	☑	☑	☑

**Table 6:** Comparison of PSNR and SSIM of IDCASR-MMF on the Test Set When the Amplification Factor Is 2/4.

Method	SET5 (×2)	Set5 (×4)	Urban100 (×2)	Urban100 (×4)	Bone FD (×2)	Bone FD (×4)
Baseline	37.10/0.9553	31.02/0.8805	30.10/0.8994	25.50/0.7566	31.10/0.9027	28.10/0.7923
Baseline + DCSE	37.62/0.9594	31.57/0.8859	30.81/0.9082	25.94/0.7689	32.18/0.9165	29.46/0.8027
Baseline + Mixed loss	37.56/0.9585	31.66/0.8883	30.77/0.9064	26.04/0.7721	32.27/0.9181	29.51/0.8079
Baseline + DC + Mixed loss	37.71/0.9598	31.90/0.8916	31.12/0.9092	26.21/0.7785	33.02/0.9255	29.95/0.8113
IDCASR-MMF	37.92/0.9602	32.26/0.8964	31.91/0.9394	26.34/0.7787	34.52/0.9437	32.87/0.8702

**Table 7:** Quantitative Evaluation Results of the Downstream Clinical Classification Task.

Method	Accuracy (%)	Precision (%)	Recall (%)	AUC
HR (Upper Bound)	93.45	93.82	92.9	0.958
Bicubic	75.8	76.45	74.22	0.805
EDSR	84.5	84.92	83.15	0.883
IDCASR-MMF	89.85	90.15	89.02	0.928

## Data Availability

The Bone Break Classifier Dataset used in this study is publicly available at https://www.kaggle.com/datasets/amohankumar/bone-break-classifier-dataset (accessed on 28 July 2026). The code supporting the findings of this study is available from the corresponding author upon reasonable request.

## Abbreviations

The following abbreviations are used in this manuscript:

CNN	Convolutional Neural Network
CT	Computed Tomography
DCSE	Dilated Convolution with Squeeze-and-Excitation
DCSEG	Dilated Convolution-Based Fusion Attention Group
GAN	Generative Adversarial Network
GAP	Global Average Pooling
HR	High-Resolution
HVS	Human Visual System
IDCASR-MMF	Improved Dilated Convolution Attention–Multiple Module Fusion
IQA	Image Quality Evaluation
LR	Low-Resolution
MISR	Medical Image Super-Resolution Reconstruction
MRI	Magnetic Resonance Imaging
MSE	Mean Squared Error
PSNR	Peak Signal-to-Noise Ratio
ReLU	Rectified Linear Unit
SE	Squeeze-and-Excitation
SR	Super-Resolution
SSIM	Structural Similarity
